# Physical Signs and Hematological and Biochemical Alterations in *Salmonella–Plasmodium* Sequentially Coinfected Albino Wistar Rats

**DOI:** 10.1155/tswj/6174005

**Published:** 2026-07-26

**Authors:** Omer Bébé Ngouateu, Raceline Gounoue Kamkumo, Paul Keilah Lunga, Ulrich Kévin Kento Temfack, Léon Daniel Kede Kede, Albert Donatien Atsamo, Marius Jaures Tsakem Nangap, Paul Désiré Dzeufiet Djomeni, Blaise Dondji, Pierre Kamtchouing

**Affiliations:** ^1^ Laboratory of Animal Physiology and Therapeutic Research, Department of Animal Biology and Physiology, University of Yaoundé I, Yaoundé, Cameroon, uy1.uninet.cm; ^2^ Laboratory of Phytobiochemistry and Medicinal Plants Studies, Department of Biochemistry, University of Yaoundé I, Yaoundé, Cameroon, uy1.uninet.cm; ^3^ Laboratory of Cellular Immunology and Parasitology, Department of Biological Sciences, Central Washington University, Ellensburg, Washington, USA, cwu.edu

**Keywords:** biochemical change, hematology, malaria, physical signs, sequential coinfection, typhoid

## Abstract

**Background:**

In endemic countries, people remain at risk of contracting both malaria and typhoid concurrently. Though experimental animal models are very reliable for understanding human diseases, the rat model for *Plasmodium–Salmonella* coinfection remains underresearched.

**Objectives:**

The present study was performed to characterize the rat model of malaria–typhoid coinfection focusing on physical, hematological, and biochemical parameters.

**Methods:**

Thirty‐four Wistar rats were distributed into five groups including normal control, immunosuppressed, *Salmonella*‐infected, *Plasmodium*‐infected, and *Salmonella–Plasmodium*‐coinfected. Physical signs, mobility, and the fecal appearance of animals were observed. *Plasmodium* load was monitored through microscopic Giemsa‐staining thin blood smears. Concurrently, *Salmonella* load was evaluated after feces culture in *Shigella–Salmonella* agar medium based on the McFarland turbidity to determine the number of colony‐forming units. Hematological and biochemical parameters were assessed using an automatic hematology analyzer and a spectrophotometer, respectively.

**Results:**

All the infected animals exhibited at least one clinical sign including piloerection, eye depigmentation, or hyperthermia. Coinfected animals showed severe anemia, leukocytosis, and thrombocytosis compared with monoinfected animals. A significant (*p* < 0.001) drop in total blood proteins and cholesterol levels was observed in infected rats, with a remarkable decrease in *Salmonella–Plasmodium*‐coinfected rats, whereas transaminase levels significantly (*p* < 0.001) increased in the same coinfected group.

**Conclusion:**

This model provides many important features of *Salmonella* and *Plasmodium* sequential coinfection and therefore may be used as a tool to better understand, diagnose, and care for human malaria and/or typhoid pathogenesis.

## 1. Introduction

Humanity′s war against infectious diseases is a long‐lasting and boring conflict, one which has not yet been won [1]. Amongst infectious diseases, there are malaria and typhoid, which are both febrile with major public health consequences in tropical and subtropical countries [2, 3]. It is revealed that in 2020, the number of malaria cases was approximately 241 million globally accounting for an estimated 627,000 deaths worldwide. Compared with the year 2019, this number represents 14 million more cases and 69,000 more deaths registered. sub‐saharan Africa region continues to bear the majority of the malaria burden with an estimated 95% of cases and 96% of all deaths in 2020 [4]. Cameroon is among the 11 countries that account for 92% of malaria infections in sub‐Saharan Africa [5]. This vector‐borne disease continues to be the main cause of death in Cameroon, and accounts for 35%–40% of all deaths, with 50% of morbidity among children under the age of five [6]. Similarly, an estimated 11–21 million typhoid fever cases with 148,000–161,000 associated deaths occurred in 2015 worldwide. In 2019, an updated modeling study estimated that 9.2 million typhoid fever cases and 110,000 deaths occurred worldwide. Cameroon is among the group of countries with incidences of more than 500 [7]. Typhoid is a systemic infectious disease whose first clinical symptoms can be headache, abdominal pain, relative bradycardia, splenomegaly, leucopenia, anorexia, persistent fever, rash, cough, constipation or diarrhea, and lymphatic enlargement [8]. If typhoid is not treated, bleeding can also be observed in more serious cases [9]. If malaria and typhoid are not diagnosed and treated early, they can contribute to death. It is reported that people in endemic areas are at risk of these two diseases concomitantly [2, 3]. A number of mechanisms explaining why malaria patients are more susceptible to *Salmonella* species bloodstream invasion have been suggested, but many critical factors pertaining to the pathogenesis of this coinfection are still to be understood [10]. Moreover, if malaria and typhoid fever are caused by very different microorganisms–one, a gram‐negative bacillus, the other, a protozoan—and transmitted via different mechanisms, they show rather similarities in their symptomatology [11]. The first description of malaria–typhoid coinfection was in the middle of the 19th century and was named typhomalarial fever by the United States Army [8], but unexpectedly, a few literature sources suggest that malaria infection is associated with *Salmonella* bacteremia [12], and many parameters linked to the pathogenesis of the coinfection are yet to be explored [10].

Experimental animal models are very relevant to understanding human pathology. Not only do they represent the first crucial step in the research of the underlying disease pathogenesis and development of new drug compounds and vaccines, but they also pave the way into modern biomedical research [13]. In rodents, *Salmonella typhimurium* induces a disease resembling the human systemic typhoid induced by *Salmonella typhi* [14]. The use of rodent *Plasmodium* species such as *Plasmodium berghei*, *P. yoelii*, and *P. chabaudi* in experimental malaria is because these species reflect many aspects of human malaria biology such as parasite life cycle stages, immune responses, and pathology. This similarity is important for controlled mechanistic studies of host–pathogen interaction and pathogenesis that would be impossible in humans [15]. Unfortunately, studies focused on a rat model of *Salmonella–Plasmodium* coinfection are scarce. Such studies are necessary for the understanding of the physiopathology and initiation of efficient therapy for both typhoid fever and malaria when they affect the same individual. The present study was carried out to begin to fill this gap. Its main goal was to characterize the *Salmonella–Plasmodium* coinfection on a Wistar rat model based on some physical signs and hematological and biochemical parameters.

## 2. Materials and Methods

All methods were conducted in compliance with relevant guidelines, regulations, and legislation.

### 2.1. Experimental Animals

Thirty‐four male albino rats (Wistar strain), aged between 8 and 10 weeks and weighing between 150 and 200 g at the beginning of the study, were used. They were bred in the animal facilities at the University of Yaoundé I. They were housed collectively in cages (four animals per cage) and given free access to standard food and tap water. Their cages were lined with wood shavings and maintained at ambient temperature with sufficient ventilation and a natural light–dark cycle.

### 2.2. Microorganisms

The *P. berghei* NK 65 strain was provided by Bei‐Resource (USA). The infected red blood cells (iRBCs) used for infection were obtained after in vivo passage in rats. For infections, aliquots containing 5 × 10^6^ iRBCs/100 *μ*L previously stored at −80°C were inoculated intraperitoneally. *S. typhimurium* used was an isolate obtained from patients at Cameroon Pasteur Centre (CPC). The purity of the isolate was assessed by culturing in *Salmonella–Shigella* (SS) agar medium. Some colonies were collected and dissolved in 10 mL of NaCl 0.9% progressively to reach a 1.0 McFarland turbidity equivalent to 3 × 10^8^ colony‐forming units (CFU)/mL. This suspension produces an absorbance between 0.16 and 0.2 at 600 nm.

### 2.3. Study design

#### 2.3.1. Animals′ distribution and disease induction

Giemsa‐stained blood smears and fecal culture in SS agar were performed for each rat before and after pathogen inoculation. Animals that tested positive during the pretest were excluded from the study. Animals considered infected were those that showed a positive result after pathogen inoculation. During the course of the study, animals that died or had incomplete data for certain parameters were excluded from the analysis of those parameters. In total, 34 animals were used and divided into five groups comprising five to eight animals each. Specifically, five animals were used for noninfected animals and height for all the infected rats. Animals were randomly distributed in experimental cages as follows: Group 1 was constituted of normal control rats; Group 2 was that of immunosuppressed control rats; Group 3 was formed with *Salmonella* monoinfected rats; Group 4 was that of *Plasmodium* monoinfected rats; Group 5 was that of *Salmonella–Plasmodium* sequentially coinfected animals.

Group 1 received distilled water per os and normal saline (NaCl 0.9%) by intraperitoneal route. Cyclophosphamide was used as an immunosuppressive substance to facilitate *Salmonella* establishment [16]. The presence of the group receiving cyclophosphamide only was to isolate coinfection effects on the measured parameters relative to groups receiving both cyclophosphamide and pathogens. Due to the existence of the coinfected group, all the animals, except those of Group 1, received, per os, cyclophosphamide (3 mg/100 g bw). Forty‐eight hours later, 1 mL of a suspension containing 3 × 10^8^ CFU was orally administered to animals of Groups 3 and 5. Nine days later, Groups 4 and 5 received through the intraperitoneal route 1 mL of an inoculum containing 1 × 10^6^ *P. berghei* iRBCs per microliter. The experiment lasted 15 days.

#### 2.3.2. Evaluation of physical parameters and morbidity and mortality rates

Animals were weighed every 2 days, and body temperature was also measured. The morbidity was evaluated based on physical criteria including reduced mobility, piloerection, eye depigmentation, bloody feces, and shivering. The number of animals that died during the experimentation was recorded and the mortality rate then estimated.

#### 2.3.3. Evaluation of Pathogen Load

For the bacterial load, the fecal material (0.1 g) of each experimental animal collected into a sterilized tube was dissolved in 5 mL of saline NaCl 0.9%. Then, 100 *μ*L of the suspension was displayed in a 90‐mm Petri dish filled with SS agar and incubated at 37°C for 24 h. The bacterial load was recorded by counting the number of *S. typhimurium* CFU, expressed per gram of fecal matter of each animal.

*Plasmodium* parasite count was determined using 10% Giemsa‐stained thin smear film and counting parasites per 100X red blood cells in three fields under a microscope with immersion oil and 100X objective. Parasitemia was expressed as the percentage of parasitized red blood cells.

#### 2.3.4. Animal Sacrifice and Sample Collection

Rats were anesthetized by the intraperitoneal injection of ketamine/Valium solution (75 mg/kg + 5 mg/kg body weight). Their consciousness was verified by testing the foot reflexes, heartbeats, and breathing movements. They were then dissected, and blood was collected by cardiac puncture. Part of the blood was put in EDTA tubes for hematological examination, while the remaining part was put into dried tubes for the evaluation of some serum biochemical markers. After sacrifice, some organs were collected and weighed. Their relative weights were calculated and expressed as the weight of the organ per 100 g of the body weight measured.

#### 2.3.5. Hematological and Biochemical Analyses

Blood parameters were evaluated using a Mindray BC3000 automatic hematology analyzer. The device uses the Coulter method for determining the number of white blood cells, red blood cells, and platelets. On the other hand, the colorimetric method determines the hemoglobin concentration. This device aspirates blood contained in EDTA tubes and automatically counts the blood cells.

Total proteins, cholesterol, and alanine aminotransaminase (ALAT) and aspartate aminotransaminase (ASAT) activities were estimated using specific colorimetric kits (BIOLAB). The absorbance was read using the spectrophotometer GENESY 20, and the level of each biochemical parameter was calculated as indicated on the respective kits.

### 2.4. Ethical Consideration

All experiments were performed in accordance with the ethical guidelines for experiments with rats. The study was approved by the Center for Research and Graduate Studies in Life, Health and Environment of the University of Yaoundé I. The ethical clearance (N°BTC‐JIRB2021‐014) was obtained from the Joint Institutional Review Board for Animal and Human Bioethics (JIRB).

### 2.5. Statistical Analyses

Data were expressed as the mean ± SEM (standard error of the mean). Statistical analysis of the results was performed through a one‐way ANOVA (analysis of variance) test followed by Tukey′s multiple comparison posttest using GraphPad Prism software Version 8.01. The difference was considered significant at *p* ≤ 0.05.

## 3. Results

### 3.1. Effect of *Plasmodium–Salmonella* Coinfection on Physical Aspect, Morbidity, and Mortality Rates of Animals

The data relating to the variation of different physical parameters as well as to the morbidity and mortality rates of rats infected by *Plasmodium* and/or *Salmonella* are summarized in Table 1. All the animals with a positive test for *Salmonella* and/or *Plasmodium* showed at least one morbidity sign. If the locomotor activity of *Salmonella*‐infected animals was less altered, *Plasmodium*‐infected animals (monoinfected and coinfected) showed a very high decrease in locomotor activity. Apart from this change, the inoculation of the germs led to a more marked morbid state in all the infected animals. Very bristly hair was observed in all coinfected animals as well as in 87.5% of *Plasmodium*‐infected rats; bloody stools, ocular pallor, and a very high mortality rate were recorded in coinfected animals. The mortality rate registered before 15 days (the end of the experiment) was 25%, 50%, and 37.5%, respectively, for *Salmonella*‐infected, *Plasmodium*‐infected, and coinfected animals.

**Table 1:** Effect of infectious status of the animal on the physical aspect of the animal and morbidity and mortality rates.

Animal groups					
Parameters	Control (*n* = 5)	Immuno‐suppressed (*n* = 5)	Salmonella Infected (*n* = 6)	Plasmodium infected (*n* = 8)	Coinfected (*n* = 8)
Piloerection	0/5	0/5	2/6	7/8	8/8
Bloody stool with mucus	0/5	0/5	4/6	1/8	6/8
Ocular pallor	0/5	0/5	1/6	8/8	8/8
Reduction in locomotion	0/5	0/5	3/6	8/8	8/8
Morbidity rate (%)	0	0	100	100	100
Mortality rate (%)	0	0	16.67	37.50	50

### 3.2. Effect of Coinfection on the Body Temperature

**Figure 1:**
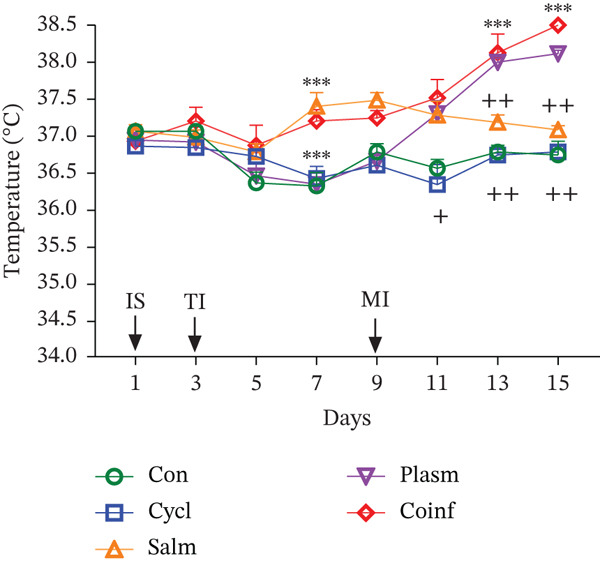
Evolution of the body temperature of animals infected by *Salmonella* and/or *Plasmodium.* Coinf: coinfected, Con: normal control, Cycl: cyclophosphamide immunosuppressed rats, IS: immunosuppression, MI: malaria induction, Plasm: *Plasmodium*‐infected rats, Salm: *Salmonella*‐infected, TI: typhoid induction. Values are expressed as mean ± SEM, *n* = 5 for noninfected and 8 for infected animals at the beginning of the test, ^++^ *p* < 0.01, ^+++^ *p* < 0.001 significant difference when compared with the coinfected group.

Figure 1 summarizes the evolution of animals′ body temperature after typhoid fever and malaria induction. It was observed that *Plasmodium*–*Salmonella* coinfection led to a significant (*p* < 0.05) increase in body temperature from Day 2 postinoculation of *P. berghei*. The temperature reached 38.6°C on Day 6 postinoculation of *P. berghei*. The *Plasmodium*‐infected group showed the same trend as the coinfected group but with a maximum of 38.1°C reached on Day 6 postinoculation. The temperature in the control and immunosuppressed groups was regular throughout the experiment and fluctuated between 36.3°C and 36.8°C.

### 3.3. Impact of *Salmonella* and *Plasmodium* Coinfection on Body Weight and Relative Weight of Some Organs

**Figure 2:**
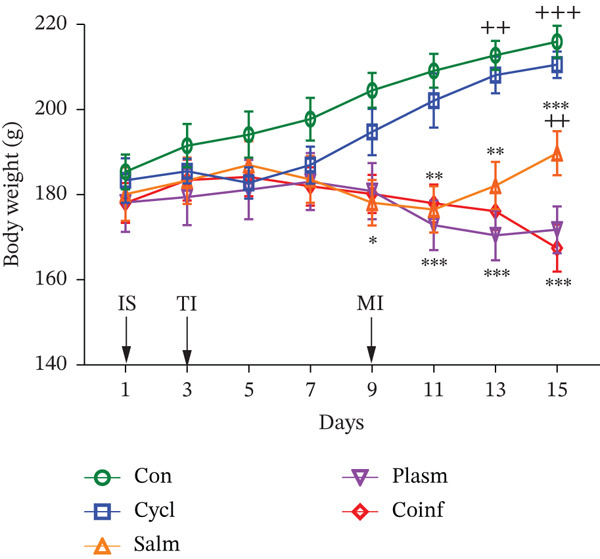
Body weight evolution of the *Salmonella*‐ and/or *Plasmodium*‐infected rats. Coinf: coinfected, Con: normal control, Cycl: cyclophosphamide immunosuppressed rats, IS: immunosuppression, MI: malaria induction, Plasm: *Plasmodium*‐infected rats, Salm: *Salmonella-*infected rats, TI: typhoid induction. Values are expressed as mean ± SEM, *n* = 5 for noninfected and 8 for infected animals at the beginning of the test, ^+^ *p* < 0.05, ^++^ *p* < 0.01, ^+++^ *p* < 0.001 significant difference when compared with the coinfected group.

*Salmonella–Plasmodium* coinfection triggered a significant (*p* < 0.001) decrease in the body weight of animals compared with the normal control (Figure 2). Compared with the coinfected animals, the body weight of *Salmonella* monoinfected animals significantly decreased (*p* < 0.05) between Days 5 and 11, followed by an increase (*p* < 0.001) in body weight at the end of the experiment. No significant change was recorded in the body weight of *Plasmodium* monoinfected and coinfected animals.

Regarding the relative weight of organs sampled, infection led to a significant increase in the relative weight of the liver (*p* < 0.05) by 65.61%, 56.15%, and 79.49% in *Salmonella*, *Plasmodium*, and *Salmonella–Plasmodium* infection, respectively, compared with the normal control (Table 2). Likewise, it was noted an increase in the relative weight of the spleen (*p* < 0.001) of 1.48, 6.77, and 5.5 times, respectively, in *Salmonella*‐infected, *Plasmodium*‐infected, and both coinfected rats related to healthy group. As far as the kidney is concerned, the weight increased (*p* < 0.001) by 2.8, 1.32, and 1.8 times for *Salmonella*‐infected, *Plasmodium*‐infected, and coinfected animals, respectively, compared with normal rats. The brain was more affected in *Salmonella*‐infected animals but less affected in other infected rats.

**Table 2:** Influence of infectious status of the animal on the weights of some vital organs.

Organs’ relative weight (mg/100 g body weight)					
	Normal control (*n* = 5)	Immuno‐suppressed (*n* = 5)	Salmonella infected (*n* = 6)	Plasmodium infected (*n* = 8)	Coinfected (*n* = 8)
Liver	3.170 ± 0.319	2.790 ± 0.106^++^	5.250 ± 0.577 ^∗^	4.950 ± 0.231 ^∗^	5.690 ± 0.817 ^∗^
Spleen	0.486 ± 0.095	0.434 ± 0.055^+++^	0.722 ± 0.079^+++^	3.73 ± 0.044 ^∗∗∗^^/^^+++^	3.122 ± 0.436 ^∗∗∗^
Kidney	0.598 ± 0.018	0.686 ± 0.018^++^	1.242 ± 0.044 ^∗∗∗^	0.790 ± 0.037	1.084 ± 0.110 ^∗∗^
Brain	0.828 ± 0.027	0.866 ± 0.037	1.650 ± 0.019 ^∗∗^^/^^++^	0.850 ± 0.023	0.966 ± 0.079

*Note:* Values are expressed as mean ± SEM,  ^∗^ *p* < 0.05,  ^∗∗^ *p* < 0.01,  ^∗∗∗^ *p* < 0.001 significant difference when compared with the normal control, ^+^ *p* < 0.05, ^++^ *p* < 0.01, ^+++^ *p* < 0.001 significant difference when compared with the coinfected group.

### 3.4. Effects of Coinfection on Each Pathogen Load

**Figure 3:**
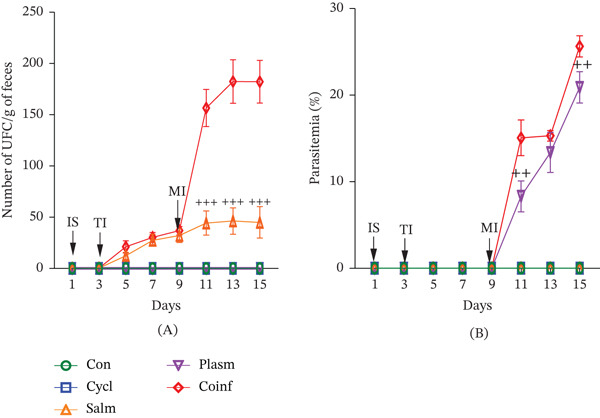
Effect of *Salmonella–Plasmodium* coinfection on (A) *Salmonella* and (B) *Plasmodium* loads. Coinf: coinfected, Con: normal control, Cycl: cyclophosphamide immunosuppressed rats, IS: immunosuppression, MI: malaria induction, Plasm: *Plasmodium*‐infected rats, Salm: *Salmonella-*infected rats, TI: typhoid induction. Values are expressed as mean ± SEM, *n* = 5 for noninfected and 8 for infected animals at the beginning of the test,  ^∗^ *p* < 0.05,  ^∗∗^ *p* < 0.01,  ^∗∗∗^ *p* < 0.001 significant difference when compared with the normal control, ^+^ *p* < 0.05, ^++^ *p* < 0.01, ^+++^ *p* < 0.001 significant difference when compared with the coinfected group.

In the *Salmonella*‐infected rats, the bacterial load in the feces increased progressively (Figure 3). It doubled almost every 2 days. In the coinfected group, before *P. berghei* inoculation, that is, 7 days after *Salmonella* inoculation, the bacterial load was 36.900 ± 4.04 CFU/g of feces. This was almost similar to 37.800 ± 6.04 CFU/g of feces observed in the *Salmonella*‐monoinfected group. Interestingly, after *P. berghei* inoculation, the number of bacterial colonies increased significantly (*p* < 0.001) to 4‐fold after 2 days and 5‐fold after 4 days postinoculation in the concerned group.

**Figure 4:**
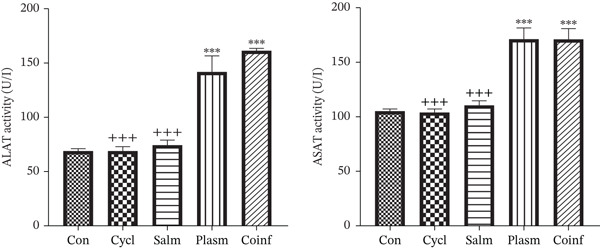
Effect of the infectious status on serum alanine aminotransferase and aspartate aminotransferase. ALAT: alanine aminotransferase, ASAT: aspartate aminotransferase, Coinf: coinfected, Con: normal control, Cycl: cyclophosphamide immunosuppressed rats, Plasm: *Plasmodium-*infected rats, Salm: *Salmonella*‐infected rats. Values are expressed as mean ± SEM, *n* = 5 for noninfected and 4 or 5 for infected animals,  ^∗^ *p* < 0.05,  ^∗∗^ *p* < 0.01,  ^∗∗∗^ *p* < 0.001 significant difference when compared with the normal control, ^+^ *p* < 0.05, ^++^ *p* < 0.01, ^+++^ *p* < 0.001 significant difference when compared with the coinfected group.

*Plasmodium*‐monoinfected rats showed a gradual increase in parasitemia that was very significant 2 days after *Plasmodium* inoculation (Figure 4). Compared with the second day postinfection, the number of parasites was 1.5‐fold and 2.5‐fold on Days 4 and 6, respectively. Coinfected rats showed a rapid increase and significantly (*p* < 0.05) high parasitemia after inoculation.

### 3.5. Impact of the Infectious Status on Hematological Parameters

The effects of infectious status on some hematological parameters are summarized in Table 3. It appears that the inoculation of *Plasmodium* to experimental animals resulted in a significant (*p* < 0.01) decrease in the number of red blood cells as well as the hemoglobin concentration, mean corpuscular hemoglobin concentration, mean corpuscular hemoglobin, hematocrit, and mean corpuscular volume in the *Plasmodium*‐infected and *Salmonella–Plasmodium*‐coinfected animals. For *Salmonella*‐infected rats, even if a significantly lower (*p* < 0.01) RBC count was observed compared with the control, this number was higher than that of *Plasmodium*‐infected and coinfected animals. On the contrary, the hemoglobin rate, the mean corpuscular hemoglobin concentration, and the mean corpuscular hemoglobin were not altered in *Salmonella*‐infected animals.

**Table 3:** Effect of infectious status on blood parameters of *Salmonella* and/or *Plasmodium* infected rats.

Blood parameters	Infectious status of the animals				
	Normal control (*n* = 5)	Immuno‐suppressed (*n* = 5)	*Salmonella*‐infected rats (*n* = 5)	*Plasmodium*‐infected rats (*n* = 5)	Coinfected animals (*n* = 4)
WBC (10^3^/mm^3^)	7.77 ± 0.31	3.32 ± 0.49^+++^	14.22 ± 1.03 ^∗^^/^^++^	18.47 ± 0.88 ^∗∗∗^	21.78 ± 2.36 ^∗∗∗^
LYM (10^3^/mm^3^)	1.95 ± 0.18	0.94 ± 0.22^+++^	6.52 ± 0.71 ^∗∗^^/^^+^	8.42 ± 0.84 ^∗∗∗^	9.45 ± 1.01 ^∗∗∗^
MON (10^3^/mm^3^)	0.4 ± 0.07	0.5 ± 0.2	1.36 ± 0.61	1.2 ± 0.36	1.62 ± 0.38
GRA (10^3^/mm^3^)	3.47 ± 0.35	0.6 ± 0.16 ^∗∗^/ ^+++^	4.56 ± 0.72	6.26 ± 1.35	5.7 ± 0.45
RBC (10^6^/mm^3^)	5.26 ± 0.25	5.53 ± 0.17^+++^	4.88 ± 0.43^++^	3.54 ± 0.28 ^∗^	3.13 ± 0.13 ^∗∗^
HGB (g/dl)	12.78 ± 0.40	14.58 ± 0.39^+++^	13.48 ± 1.04^++^	9.50 ± 0.78	8.55 ± 0.58 ^∗∗^
HCT (%)	43.45 ± 1.06	42.43 ± 2.04^+^	38.04 ± 1.99	28.93 ± 2.92 ^∗^	30.78 ± 1.47 ^∗^
MCH (pg)	28.25 ± 0.64	29.98 ± 0.85^+++^	30.03 ± 0.79 ^∗∗∗^	26.75 ± 0.63	23.78 ± 0.99 ^∗∗^
MCHC (g/dl)	31.15 ± 0.29	32.35 ± 0.56	33.4 ± 0.49^+^	27.98 ± 1.58	28.03 ± 1.65
MCV (fl)	87.5 ± 0.64	86.5 ± 0.64^+++^	84.4 ± 3.14^+^	70.33 ± 1.20 ^∗∗∗^	67.25 ± 1.25 ^∗∗∗^
PLT (10^3^/mm^3^)	330.5 ± 12.81	427.5 ± 67.31^++^	638.8 ± 40.48 ^∗∗^^/^^+^	719.3 ± 74.78 ^∗^	853.5 ± 32.17 ^∗∗^

*Note:* Values are expressed as mean ± SEM,  ^∗^ *p* < 0.05,  ^∗∗^ *p* < 0.01,  ^∗∗∗^ *p* < 0.001 significant difference when compared with the normal control, ^+^ *p* < 0.05, ^++^ *p* < 0.01, ^+++^ *p* < 0.001 significant difference when compared with the coinfected group.

Abbreviations: GRA: granulocytes, HCT: hematocrit, HGB: hemoglobin rate, LYM: lymphocyte, MCH: mean corpuscular hemoglobin, MCHC: mean corpuscular hemoglobin concentration, MCV: mean corpuscular volume, MON: monocyte, PLT: platelet, RBC: red blood cell, WBC: white blood cell.

As far as the number of WBC is concerned, the infectious status led to a significant increase in their number in all the infected groups but a greater significance (*p* < 0.001) in the *Plasmodium*‐infected and coinfected animals. The WBC count of immunosuppressed rats was obviously very low. As compared with normal animals, the number of WBCs was 1.8, 2.4, and 2.8 times higher, respectively, for *Salmonella*‐infected, *Plasmodium*‐infected, and coinfected rats. The increase concerned all WBC types (lymphocytes, monocytes, and granulocytes). Whatever the case, coinfected animals showed a higher number of all WBCs compared with other groups.

The number of platelets also increased significantly in infected animals compared with the control. The increase was 1.9, 2.2, and 2.6 times for *Salmonella*‐infected, *Plasmodium*‐infected, and coinfected animals, respectively.

### 3.6. Influence of the Infection Status on Blood Transaminases

The effect of *Salmonella* and *Plasmodium* coinfection on the activities of serum ALAT and ASAT is depicted in Figure 4. This figure shows that both infections led to a significant increase (*p* < 0.001) in the ALAT and ASAT levels in *Plasmodium*‐infected and coinfected groups at respective rates of 51.38% and 57.25% for ALAT and 38.56% and 38.53% for ASAT compared with the normal control. A significantly low (*p* < 0.001) activities of ALAT and ASAT were noted in the immunosuppressed and *Salmonella*‐infected groups at the respective rates of 8.28% and 53.93% for ALAT and 39.13% and 35.39% for ASAT compared with coinfected rats. No significant difference in transaminase activity was observed in the *Plasmodium*‐monoinfected group compared with coinfected animals.

### 3.7. Effect of Coinfection on Serum Cholesterol and Total Protein Concentration

**Figure 5:**
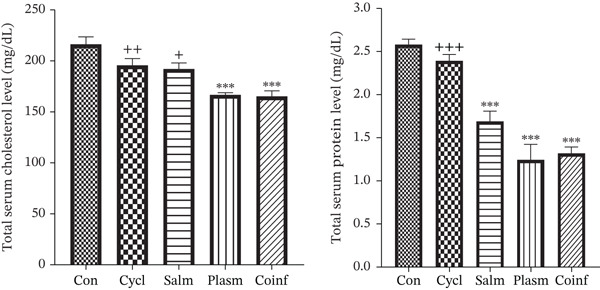
Effect of the infectious status on serum total cholesterol and protein. Coinf: coinfected, Con: normal control, Cycl: cyclophosphamide immunosuppressed rats, Plasm: *Plasmodium*‐infected rats, Salm: *Salmonella*‐infected rats. Values are expressed as mean ± SEM, *n* = 5 for noninfected and 4 or 5 for infected animals,  ^∗^ *p* < 0.05,  ^∗∗^ *p* < 0.01,  ^∗∗∗^ *p* < 0.001 significant difference when compared with the normal control, ^+^ *p* < 0.05, ^++^ *p* < 0.01, ^+++^ *p* < 0.001 significant difference when compared with the coinfected group.

The effect of *Plasmodium*–*Salmonella* coinfection on serum cholesterol concentration is summarized in Figure 5A. It appears that the inoculation of bacterial and protozoan strains resulted in a significantly low level (*p* < 0.001) of serum cholesterol in all the infected groups. For *Salmonella-*monoinfected, *Plasmodium-*monoinfected, and coinfected animals, serum cholesterol levels were significantly lower than that of the normal control by 11.16% (p < 0.05), 22.95% (p < 0.001), and 23.58% (p < 0.001) respectively. As far as the serum protein is concerned (Figure 5B), all the infected animals showed a significantly low level. This was very pronounced in *Salmonella–Plasmodium*‐coinfected animals.

## 4. Discussion

The present study aimed to evaluate some physical, hematological, and biochemical damages induced by *Salmonella* and *Plasmodium* when the two pathogens sequentially infected the same organism. Although in nature coinfection may result from simultaneous or sequential infection, it is more likely to result from sequential infection rather than simultaneous infection. A case where *Salmonella* infection occurred prior to *Plasmodium* was considered in the present study. Previous coinfected models have made use of mice such as BALB/c or LACA mice and C57BL/6 mice [17]. However, the rat model is advantageous in that it furnishes more blood samples for various clinical analyses. In the present study, the cyclophosphamide‐only group clearly revealed the effect of coinfection on the studied parameters when compared with the groups that received both cyclophosphamide and pathogens. *Salmonella* and *Plasmodium* infections are both febrile [18]. The febrile state of these infections was confirmed in the present experimental study. The body temperature of the *Salmonella–Plasmodium*‐coinfected animals was higher than that of controls and monoinfected animals. It is reported that febrile response is mediated by endogenous pyrogens (cytokines) in response to exogenous pyrogens, primarily microorganisms or their direct products (toxins). Moreover, if in gram‐positive bacteria the main pyrogen is the peptidoglycan, the pyrogenicity of gram‐negative bacteria like *Salmonella* is due to a heat‐stable factor. The active components of this factor are lipid and carbohydrate (lipopolysaccharide), which are the major components of the outer membrane of these bacteria [19]. Moreover, it is known that malaria‐specific metabolite hemozoin mediates the release of several potent endogenous pyrogens (TNF, MIP‐1 alpha, and MIP‐1 beta) and altered thermoregulation [20]. Kubata et al. [21] reported that *Plasmodium* produces prostaglandins that are pyrogenic, somnogenic, and immunosuppressive substances. In the present study, when *Salmonella* and *Plasmodium* are both present, the higher temperature observed in coinfected rats when compared with each infection alone may be justified by the additive effect of their pyrogens. Regarding body weight, it rather decreased, and the decrease was very pronounced in coinfected rats. It was previously reported that *Plasmodium* or *Salmonella* infection can cause nausea, vomiting, abdominal pain, diarrhea, fatigue, and fever [22]; the corollary of which is the body weight loss of animals. Hypoproteinemia observed in rats also corroborated this weight loss. In fact, hypoproteinemia can present a spectrum of symptoms including muscle wasting, hair loss, and fatigue. Hypoproteinemia occurs principally due to decreases in albumin and immunoglobulin [23]. Plasma proteins level depends on the relation between the synthesis, destruction, and distribution of proteins in tissues [24]. Reduction in blood protein level may be due to their loss at the intestinal level as a consequence of *Salmonella* infection. In fact, protein‐losing enteropathy is a medical condition that is characterized by loss of protein, vitamins, and trace elements into the intestines, which can be complicated by various diseases, namely, *Salmonella* infection [20]. Hypoproteinemia accentuation observed in the present study may be also due to the use of plasma proteins by *Plasmodium* because this parasite, for its growth, acquires nutrients from plasma [25, 26].

In the present study, the coinfected rats showed higher bacterial load and higher parasitemia compared, respectively, to that of *Salmonella*‐ and *Plasmodium-*monoinfected rats. Malaria is thought to increase susceptibility to bacteremia by impairment of gastrointestinal barrier defenses and impairment of immune response [27]. Products of *Plasmodium* spp–induced hemolysis cause the production of heme oxygenase‐1 (HO‐1), which is efficient in iron recycling while hindering the harmful prooxidant effect resulting from the heme group [10]. HO‐1 induction has been shown to protect against infectious, inflammatory, and hypoxic‐ischemic insults in mice and has been linked to modulation of malarial pathogenesis and sickle cell disease [28]. Consequently, malarial hemolysis counteracts neutrophil development required to build an adequate oxidative defense mechanism, leading to the formation of *Salmonella* and other gram‐negative bacteria reservoirs. Concerning macrophages, this hemolysis affects their iron homeostasis. In fact, the heme‐liberated iron penetrates these macrophages, where it is an essential nutrient for *Salmonella* and like pathogens. Thus, the retention of iron by macrophages in the case of malaria promotes bacterial replication [10]. This may explain the higher load of *Salmonella* in coinfected rats compared with animals with only *Salmonella*. Although direct evidence that *Salmonella* infection enhances *Plasmodium* load is scarce, our findings showing high parasitemia in coinfected rats may be explained by *Salmonella*‐induced dysregulation.

In humans, the pathophysiology of typhoid fever involves several stages beginning with an asymptomatic incubation period of 7–14 days during which macrophage proliferation can be registered throughout the reticuloendothelial system. During the first week of symptomatic disease, progressive rise in temperature with bacteremia is observed. In the second week, rose spots, abdominal pain, and splenomegaly are evident. Following the third week, complications like perforation, pneumonia, and encephalopathy [18] occur. In more serious cases of typhoid, hemorrhage can be observed [9–18]. In the present study, this complication was observed in *Salmonella*‐infected and coinfected animals, which showed feces with blood and mucus. *Salmonella*‐monoinfected animals showed a drop in the number of red blood cells, but the hemoglobin concentration was not altered, be it at the whole blood level or at the corpuscular level. This is evident because Shilpa et al. [18] previously reported that typhoid fever shows normochromic normocytic anemia. But in the case of *Salmonella* infection or *Salmonella–Plasmodium* coinfection, the results of the present study showed a severe hypochromic anemia with a decrease in hematocrit. Working on mice, Kaye et al. [29] reported that there was no consistent difference in mean hematocrit values between animals with both *Salmonella* and *Plasmodium* infection and those with only *Plasmodium*. Therefore, they suggested that the decrease in survival time was related to the enhancement of *Salmonella* infection.

Anemia, leukopenia, and thrombocytopenia are hematological abnormalities commonly observed in typhoid [30, 31] and malaria patients [30]. The occurrence of cytopenia is more often due to bone marrow suppression and hemophagocytosis [30]. Recent innovations in multi‐omics profiling have successfully mapped the biological and phenotypic diversity of platelets. It is reported that platelets′ role is not only in hemostasis but also in the regulation of inflammation and immune responses during infection. They participate in the immune response by direct recognition of pathogen/damaged‐associated molecular patterns. Concurrently, activated platelets liberate a vast repertoire of immunomodulatory mediators to coordinate the precise recruitment of granulocytes, monocytes, and lymphocytes. By bridging adhesion molecules—predominantly selectins and integrins—with localized endothelial chemokine gradients, platelets orchestrate the formation of heterotypic aggregates critical to the vascular inflammatory cascade [32]. Al Reesi et al. [33] mentioned that thrombocytopenia is relatively common in typhoid fever, with an incidence up to 26% in children. Previously, Yildirim et al. [34] reported a severe case of thrombocytopenia in a 10‐year‐old boy. Thrombocytopenia has been classified as a marker of severity in typhoid fever and indicates a high risk for development of complications. In the present study, thrombocytosis was rather observed in all infected groups with a severe increase in platelet numbers in *Salmonella–Plasmodium*‐coinfected rats. Knowledge of the response of thrombocytes to different human infections is limited. Incrimination of thrombocytosis in acute infections is not frequent because megakaryopoiesis is inhibited at the acute infection stage by many factors like bacterial endotoxin, transforming growth factor *β*, and tumor necrosis factor. Contrarily, chronic infections are most often related to reactive thrombocytosis [35]. Even if malaria and enteric fever did not show any significant association with thrombocytosis in a study performed by Thakur and Kattel [36], these authors found an association with thrombocytosis and febrile illnesses in children (3 months to 14 years). The present study is in accordance with the authors showing thrombocytosis in *Salmonella* and/or *Plasmodium* infections in a rat model. Both *Plasmodium* and *Salmonella* infections trigger strong innate immune activation. Interleukin‐6 upregulates hepatic thrombopoietin production, which stimulates megakaryocyte proliferation and increased platelet output. In coinfection, additive cytokine signaling can tip the balance toward thrombocytosis once early consumption/sequestration abates [37, 38]. The degree of thrombocytosis is therefore related to the severity and the chronicity of single‐agent infection or coinfection.

Previous works incriminated macrophage and neutrophil dysfunctions in promoting *Salmonella* bacteremia, as they are fundamental phagocytic cells that engulf bacteria [10]. Neutrophils participate in the defense response against the malaria parasite via phagocytosis and reactive oxygen species (ROS) production. But they might also contribute to the pathogenesis of malaria by releasing toxic granules and neutrophil extracellular traps (NETs). Intriguingly, *Plasmodium* species, by inhibiting the antimicrobial function of neutrophils, make malaria patients more susceptible to opportunistic *Salmonella* infections [39]. Leukocytosis has been previously reported in mice infected with *P. berghei* [40]. *Salmonella* and/or *Plasmodium* rats also showed leukocytosis, which was very pronounced in coinfected animals. Neutrophils in this case might release toxic granules and NETs to exacerbate malaria pathogenesis. *Plasmodium* might hamper neutrophil function and allow the multiplication of *Salmonella*. This mechanism needs to be deeply investigated.

Hepato‐splenomegaly was observed in all infected rats. The increased size of the spleen and the liver could be the result of increased stimulation of phagocytic activity in these organs and extramedullary erythropoiesis [39]. The ASAT and ALAT activities were very high in coinfected animals indicating a possible alteration of the integrity of the hepatic barrier. This leads to the liberation of the hepatic enzymes into the blood circulation. The increase in ALAT and ASAT activities revealed the impairment of liver function [41]. Although the literature showing specific blood cholesterol level changes directly caused by *Salmonella* infection alone is poorly documented, several plausible hypotheses exist regarding the pathophysiology of lipid profile changes in *Plasmodium* infection [42]. Parasites, such as *Plasmodium,* are critically dependent on host nutrients. *Plasmodium* cannot synthesize cholesterol, and its source of lipid is from the host [43, 44]. A previous study indicates that malaria and/or typhoid induce a decrease. *Salmonella* and/or *Plasmodium* infections elicited decreased low‐density lipoprotein, very low‐density lipoprotein cholesterol, and phospholipids [44]. As observed in the present work, typhoid causes a slight drop in blood total cholesterol. The markable drop in coinfected animals is likely due to *Plasmodium* infection. All the complications that occurred in coinfected rats accelerated their death. This is in accordance with previous studies, since it has been reported that concurrent infection in mice with *S. typhimurium* and *P. berghei* is more rapidly fatal than either infection alone [29]. This idea is also corroborated in an experimental rat model and may provide insights into human *Salmonella–Plasmodium* coinfection.

## 5. Conclusion

The present study demonstrates that sequential *Salmonella–Plasmodium* coinfection in rats is associated with more severe clinical manifestations than either infection alone. Coinfected animals exhibited marked clinical, hematological, and biochemical alterations, including weight loss, organ enlargement, anemia, leukocytosis, thrombocytosis, elevated liver enzyme levels, and increased pathogen burden. Compared with single infections, coinfection resulted in greater disease severity and earlier mortality. These findings underscore the importance of considering the impact of coinfections on disease progression and host health.

## Author Contributions

Conceptualization: OBN, BD, PK; Formal analysis: OBN, RGK, PKL, UKKT, LDKK, ADA, PDDD; Investigation: OBN, RGK, PKL, UKKT, LDKK, MJTN, PDDD; Methodology: OBN, RGK, PKL, UKKT, ADA, MJTN; Writing—original draft, OBN, RGK, PKL, ADA, BD, PDDD; Writing—review and editing, OBN, RGK, UKKT, ADA, PDDD, BD, PK.

## Funding

No funding was received for this manuscript.

## Disclosure

All authors have read and approved the final version of the manuscript. Omer Bébé Ngouateu Ngouateu had full access to all of the data in this study and takes complete responsibility for the integrity of the data and the accuracy of the data analysis.

## Conflicts of Interest

The authors declare no conflicts of interest.

## Data Availability

The data that support the findings of this study are available from the corresponding author upon reasonable request.
